# *catena*-Poly[[[triaqua­sulfatozinc(II)]-μ-3,3′-bis­(3-pyrid­yl)-1,1′-(*m*-phenyl­ene)diurea] methanol solvate monohydrate]

**DOI:** 10.1107/S1600536810009268

**Published:** 2010-03-17

**Authors:** N. N. Adarsh, Parthasarathi Dastidar

**Affiliations:** a Department of Organic Chemistry, Indian Association for the Cultivation of Science, 2A & 2B Raja S C Mullick Road, Jadavpur, Kolkata 700 032, India

## Abstract

In the title coordination polymer, {[Zn(SO_4_)(C_18_H_16_N_6_O_2_)(H_2_O)_3_]·CH_3_OH·H_2_O}_*n*_, the Zn^2+^ ion adopts a slightly distorted *cis*-ZnN_2_O_4_ octa­hedral geometry arising from three coordinated water mol­ecules, one sulfate ion and two bridging 3,3′-bis­(3-pyrid­yl)-1,1′-(*m*-phenyl­ene)diurea (bpmpbu) ligands. The dihedral angles between the central benzene ring and two terminal pyridine rings of the bpmbpu mol­ecule are 10.58 (17) and 34.63 (16)°. In the crystal, the ligands bridge the Zn^II^ ions, thus generating a one-dimensional zigzag coordination polymer propagating in [010]. The crystal structure features extensive N—H⋯O and O—H⋯O hydrogen-bonding inter­actions.

## Related literature

For our previous work on related compounds, see: Adarsh *et al.* (2008 ▶, 2009 ▶),
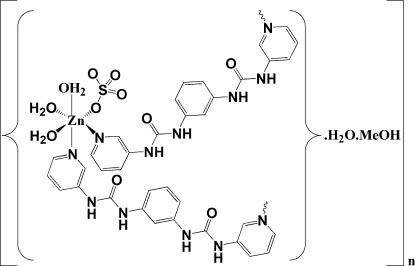


## Experimental

### 

#### Crystal data

[Zn(SO_4_)(C_18_H_16_N_6_O_2_)(H_2_O)_3_]·CH_4_O·H_2_O
*M* *_r_* = 613.90
Monoclinic, 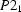

*a* = 6.4831 (7) Å
*b* = 19.265 (2) Å
*c* = 9.7062 (11) Å
β = 98.848 (2)°
*V* = 1197.8 (2) Å^3^
*Z* = 2
Mo *K*α radiation
μ = 1.19 mm^−1^
*T* = 100 K
0.28 × 0.22 × 0.12 mm

#### Data collection

Bruker APEXII CCD diffractometer
Absorption correction: multi-scan (*SADABS*; Bruker, 2006 ▶) *T*_min_ = 0.732, *T*_max_ = 0.871
5989 measured reflections
3968 independent reflections
3823 reflections with *I* > 2σ(*I*)
*R*_int_ = 0.025

#### Refinement

*R*[*F* ^2^ > 2σ(*F* ^2^)] = 0.031
*wR*(*F* ^2^) = 0.068
*S* = 1.02
3968 reflections
369 parameters
1 restraint
H atoms treated by a mixture of independent and constrained refinement
Δρ_max_ = 0.54 e Å^−3^
Δρ_min_ = −0.26 e Å^−3^
Absolute structure: Flack (1983 ▶), 1782 Friedel pairs
Flack parameter: 0.049 (10)

### 

Data collection: *APEX2* (Bruker, 2006 ▶); cell refinement: *SAINT* (Bruker, 2006 ▶); data reduction: *SAINT*; program(s) used to solve structure: *SHELXS97* (Sheldrick, 2008 ▶); program(s) used to refine structure: *SHELXL97* (Sheldrick, 2008 ▶); molecular graphics: *Mercury* (Macrae *et al.*, 2008 ▶); software used to prepare material for publication: *SHELXL97*, *publCIF* (Westrip, 2010 ▶) and *PLATON* (Spek, 2009 ▶).

## Supplementary Material

Crystal structure: contains datablocks I, global. DOI: 10.1107/S1600536810009268/hb5311sup1.cif

Structure factors: contains datablocks I. DOI: 10.1107/S1600536810009268/hb5311Isup2.hkl

Additional supplementary materials:  crystallographic information; 3D view; checkCIF report

## Figures and Tables

**Table 1:** Selected bond lengths (Å)

Zn1—N1	2.116 (3)
Zn1—N23^i^	2.126 (3)
Zn1—O33	2.062 (2)
Zn1—O31	2.149 (3)
Zn1—O32	2.158 (3)
Zn1—O27	2.217 (2)

Symmetry code: (i) 
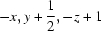
.

**Table 2:** Hydrogen-bond geometry (Å, °)

*D*—H⋯*A*	*D*—H	H⋯*A*	*D*⋯*A*	*D*—H⋯*A*
N7—H7⋯O29^ii^	0.86	2.14	2.954 (4)	157
N10—H10⋯O29^ii^	0.86	2.25	3.044 (4)	154
N17—H17⋯O28^iii^	0.86	2.05	2.875 (4)	160
N20—H20⋯O30^iii^	0.86	2.08	2.922 (4)	164
O35—H35⋯O34	0.82	2.09	2.893 (4)	168
O32—H32*A*⋯O27^iv^	0.85 (4)	2.09 (4)	2.942 (3)	179 (4)
O31—H31*A*⋯O9^iv^	0.81 (4)	2.09 (4)	2.899 (4)	173 (4)
O33—H33*A*⋯O29^iv^	0.85 (4)	1.87 (4)	2.714 (4)	170 (4)
O34—H34*A*⋯O9	0.90 (5)	2.08 (5)	2.861 (4)	146 (4)
O32—H32*B*⋯O19^v^	0.73 (4)	2.08 (4)	2.774 (3)	158 (5)
O31—H31*B*⋯O34	0.79 (4)	2.11 (4)	2.887 (4)	168 (4)
O33—H33*B*⋯O28	0.82 (4)	1.84 (4)	2.633 (3)	162 (4)
O34—H34*B*⋯O30	0.98 (5)	1.90 (5)	2.748 (4)	144 (4)

Symmetry codes: (ii) 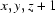; (iii) 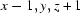; (iv) 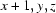; (v) 
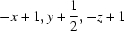
.

**Fig. 1.**
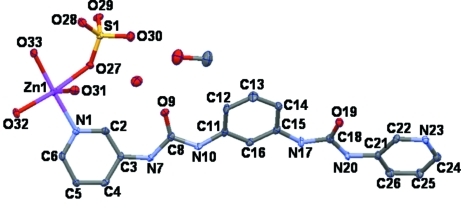
Fragment of the structure of (I) with displacement ellipsoids drawn at 50% probability level

**Fig. 2.**
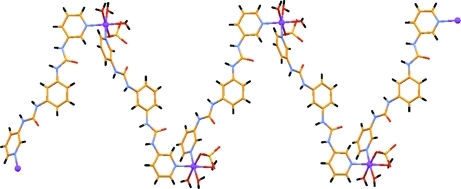
1D zig-zag coordination polymer in (I).

**Fig. 3.**
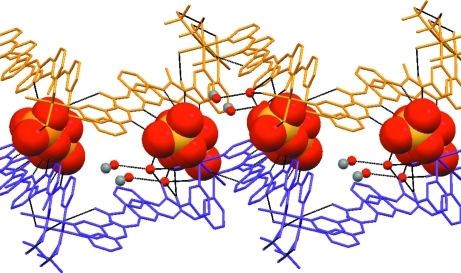
Parallel packing of 1D zigzag chains in (I) (shown in orange and purple colour) displaying various hydrogen bonding (dotted lines) intearctions (dotted lines).

## supplementary crystallographic information

### Comment

As part of our ongoing studies of functional coordination polymers (CPs) and metal-organic frameworks (MOF) (Adarsh *et al.*,  2008, 2009), we now report a coordination polymer derived from a newly synthesized bis-pyridyl-bis-urea ligand *N*,*N*'-bis(3-pyridyl)meta-phenylene-bis-urea (BPMPBU). Suitable single crystals of [{Zn(H_2_O)_3_(SO_4_)(µ-BPMPBU)}.H_2_O.MeOH]_∞_, (I), were obtained by layering an methanolic solution of BPMPBU over aqueous solution of ZnSO_4_ in 1:2 metal–ligand ratio (see experimental). It was crystallized in the monoclinic non centrosymmetric space group P2_1_. In the asymmetric unit, the metal center Zn(II) is found to be hexacoordinated; while euqatorial positions are occupied by pyridyl N atoms of the ligand and water molecules, the apical positions are coordinated by O atoms of water and sulfate. The ORTEP diagram of 1 with 50% probability is given in Fig. 1. The metal center Zn(II) displayed a distorted octahedral geometry [N—Zn—N = 94.72?(12)°; O—Zn—N = 87.4)(12)-93.83 (10)°; O—Zn—O = 87.01 (11)-91.12 (12)°]. In the crystal structure, the ligand BPMPBU which displayed *syn-syn-syn* conformation (scheme 1) coordinated to the adjacent Zn(II) metal centres via pyridyl N atoms leading to the formation of a 1D zigzag polymeric chain (Fig. 2). The polymeric chains are further packed in parallel fashion sustained by N–H···O hydrogen bonding interactions involving sulfate and the urea moieties [N···O = 2.875 (4)–3.044 (4) Å; N–H···O =154–164°]. Both the lattice included water and MeOH are occupied within the interstitial space of the chains and are involved in O—H···O hydrogen bonding interactions [O···O = 2.633 (3)–2.942 (3) Å; O–H···O] (Fig. 3).

### Experimental

3-Aminopyridine (1 g, 10.6 mmol) and triethylamine (3 ml) were stirred under nitrogen in 150 ml of anhydrous dichloromethane in ice cold condition for 20 min. To this stirring solution, triphosgene (1.57 g, 5.3 mmol) was added and kept stirring for another 20 min at 0°C and to the resultant solution, meta-phenylenediamine (574 mg, 5.3 mmol) in 10 ml of dichloromethane was added dropwise. White precipitate was obtained after 24 hour of stirring at room temperature. The precipitate was filtered, air dried, treated with 5% NaHCO_3_ solution and washed with distilled water. Pale yellow coloured microcrystalline material of the BPMPBU ligand was obtained (880 mg, 55% yield) after recrystallization from H_2_O/MeOH (1 : 2 v/v) (880 mg, 55% yield) . mp 265-267°C Anal. data calc. for C_18_H_16_N_6_O_2_: C, 62.06; H, 4.63; N, 24.12. Found: C, 61.86, H, 4.24, N, 24.32. ^1^H NMR (300 MHz, DMSO-d6): δ = 7.09-7.12 (2*H*, d, J = 9 Hz, Ar—H); 7.23-7.18 (1*H*, t, J = 9, 9 Hz, Ar—H); 7.34-7.30 (2*H*, dd, J = 6, 9 Hz, Py—H); 7.70 (1*H*, s, Ar—H); 7.96-7.93 (2*H*, d, J = 9 Hz, Py—H); 8.20-8.19 (2*H*, d, J = 3 Hz, Py—H); 8.62 (2*H*, s, Py—H); 8.78 (2*H*, s, urea N—H); 8.87 (2*H*, s, urea N—H). HRMS(ESI) calcd for C_18_H_16_N_6_O_2_ 349.14; found: [M + Na]+ 349.12 F T—IR (KBr, cm-1): 3346 (s, N–H stretch), 3242 (s, O–H stretch), 3059, 3022 (s, aromatic C–H stretch), 2908, 2827 (s, aliphatic C–H stretch), 1710, 1647 (s, urea C=O stretch), 1641 (s, urea N–H bend), 1608, 1595, 1535, 1485, 1425, 1421, 1408, 1327, 1294, 1209, 1186, 1122, 1103, 854, 786, 767, 734, 700, 626, 551.

The title compound was synthesized by layering a methanolic solution of BPMPBU (40 mg, 0.1149 mmol) over an aqueous solution of ZnSO_4_.7H_2_O (16.5 mg, 0.0575 mmol). After three days, colourless blocks of (I) were obtained (yield = 20 mg, 57 %). Anal. data calc. for C_19_H_28_N_6_O_11_SZn: C, 37.17; H, 4.60; N, 13.69, found: C, 37.12, H, 4.28, N, 13.66. F T—IR (KBr, cm-1): 3323 (b, N–H stretch), 3219 (b, O–H stretch), 3090 (b, aromatic C–H stretch), 1693 (s, urea C=O stretch), 1610 (s, urea N–H bend), 1589, 1570, 1552, 1496, 1483, 1431, 1330, 1294, 1217, 1193, 1130, 1089, 1066, 1035, 945, 900, 850, 819, 775, 704.

### Refinement

Whenever possible, the hydrogen atoms were located on a difference Fourier map and refined. In other cases, the hydrogen atoms were geometrically fixed. The positional parameters of hydrogen atoms of the water molecules and methanol molecule were refined with the constraint U_iso_(H) = 1.2 or 1.5U_eq_(carrier) applied.

### Crystal data

**Table:**

[Zn(SO_4_)(C_18_H_16_N_6_O_2_)(H_2_O)_3_]·CH_4_O·H_2_O	*F*(000) = 636
*M**_r_* = 613.90	*D*_x_ = 1.702 Mg m^−^^3^
Monoclinic, *P*2_1_	Mo *K*α radiation, λ = 0.71073 Å
Hall symbol: P 2yb	Cell parameters from 144 reflections
*a* = 6.4831 (7) Å	θ = 2.3–26.0°
*b* = 19.265 (2) Å	µ = 1.19 mm^−^^1^
*c* = 9.7062 (11) Å	*T* = 100 K
β = 98.848 (2)°	Block, colourless
*V* = 1197.8 (2) Å^3^	0.28 × 0.22 × 0.12 mm
*Z* = 2	

### Data collection

**Table:**

Bruker APEXII CCD diffractometer	3968 independent reflections
Radiation source: fine-focus sealed tube	3823 reflections with *I* > 2σ(*I*)
graphite	*R*_int_ = 0.025
Detector resolution: 3 pixels mm^-1^	θ_max_ = 25.0°, θ_min_ = 2.1°
φ and ω scans	*h* = −7→6
Absorption correction: multi-scan (*SADABS*; Bruker, 2006)	*k* = −22→22
*T*_min_ = 0.732, *T*_max_ = 0.871	*l* = −7→11
5989 measured reflections	

### Refinement

**Table:**

Refinement on *F*^2^	Secondary atom site location: difference Fourier map
Least-squares matrix: full	Hydrogen site location: inferred from neighbouring sites
*R*[*F*^2^ > 2σ(*F*^2^)] = 0.031	H atoms treated by a mixture of independent and constrained refinement
*wR*(*F*^2^) = 0.068	*w* = 1/[σ^2^(*F*_o_^2^) + (0.0188*P*)^2^] where *P* = (*F*_o_^2^ + 2*F*_c_^2^)/3
*S* = 1.02	(Δ/σ)_max_ = 0.001
3968 reflections	Δρ_max_ = 0.54 e Å^−^^3^
369 parameters	Δρ_min_ = −0.26 e Å^−^^3^
1 restraint	Absolute structure: Flack (1983), 1782 Friedel pairs
Primary atom site location: structure-invariant direct methods	Flack parameter: 0.049 (10)

### Special details

**Table:**

Geometry. All esds (except the esd in the dihedral angle between two l.s. planes) are estimated using the full covariance matrix. The cell esds are taken into account individually in the estimation of esds in distances, angles and torsion angles; correlations between esds in cell parameters are only used when they are defined by crystal symmetry. An approximate (isotropic) treatment of cell esds is used for estimating esds involving l.s. planes.
Refinement. Refinement of *F*^2^ against ALL reflections. The weighted *R*-factor wR and goodness of fit *S* are based on *F*^2^, conventional *R*-factors *R* are based on *F*, with *F* set to zero for negative *F*^2^. The threshold expression of *F*^2^ > σ(*F*^2^) is used only for calculating *R*-factors(gt) etc. and is not relevant to the choice of reflections for refinement. *R*-factors based on *F*^2^ are statistically about twice as large as those based on *F*, and *R*- factors based on ALL data will be even larger.

### Fractional atomic coordinates and isotropic or equivalent isotropic displacement parameters (Å^2^)

**Table:**

	*x*	*y*	*z*	*U*_iso_*/*U*_eq_	
Zn1	1.01818 (5)	0.83531 (2)	0.15530 (4)	0.01276 (10)	
S1	0.59561 (13)	0.76119 (4)	−0.03921 (8)	0.01383 (19)	
N1	0.9621 (4)	0.84335 (19)	0.3638 (3)	0.0137 (6)	
C2	0.7909 (5)	0.81484 (16)	0.4027 (3)	0.0159 (8)	
H2	0.6967	0.7916	0.3365	0.019*	
C3	0.7500 (5)	0.81905 (15)	0.5399 (3)	0.0133 (8)	
C4	0.8923 (5)	0.85448 (16)	0.6356 (4)	0.0157 (8)	
H4	0.8676	0.8592	0.7270	0.019*	
C5	1.0682 (5)	0.88257 (18)	0.5975 (4)	0.0159 (8)	
H5	1.1653	0.9056	0.6622	0.019*	
C6	1.0987 (5)	0.87587 (18)	0.4585 (4)	0.0164 (8)	
H6	1.2183	0.8947	0.4315	0.020*	
N7	0.5748 (4)	0.79042 (15)	0.5860 (3)	0.0174 (7)	
H7	0.5445	0.8065	0.6631	0.021*	
C8	0.4459 (5)	0.73982 (18)	0.5225 (4)	0.0147 (8)	
O9	0.4570 (4)	0.71652 (12)	0.4060 (2)	0.0177 (5)	
N10	0.3052 (5)	0.71747 (15)	0.6036 (3)	0.0165 (6)	
H10	0.3019	0.7402	0.6796	0.020*	
C11	0.1645 (5)	0.66159 (17)	0.5776 (3)	0.0138 (7)	
C12	0.1858 (6)	0.60887 (19)	0.4839 (4)	0.0185 (8)	
H12	0.2956	0.6091	0.4325	0.022*	
C13	0.0411 (6)	0.5561 (2)	0.4683 (4)	0.0248 (9)	
H13	0.0552	0.5208	0.4051	0.030*	
C14	−0.1251 (6)	0.55336 (19)	0.5430 (4)	0.0193 (9)	
H14	−0.2232	0.5179	0.5282	0.023*	
C15	−0.1405 (5)	0.60492 (17)	0.6399 (3)	0.0145 (7)	
C16	0.0004 (5)	0.65929 (17)	0.6551 (3)	0.0142 (7)	
H16	−0.0144	0.6948	0.7178	0.017*	
N17	−0.2982 (4)	0.60722 (14)	0.7258 (3)	0.0164 (6)	
H17	−0.3135	0.6464	0.7656	0.020*	
C18	−0.4296 (5)	0.55496 (17)	0.7538 (4)	0.0144 (8)	
O19	−0.4347 (4)	0.49686 (12)	0.7017 (2)	0.0166 (5)	
N20	−0.5554 (4)	0.57575 (15)	0.8485 (3)	0.0154 (6)	
H20	−0.5270	0.6156	0.8867	0.018*	
C21	−0.7245 (6)	0.54001 (18)	0.8903 (4)	0.0144 (7)	
C22	−0.7849 (5)	0.47290 (18)	0.8489 (3)	0.0140 (7)	
H22	−0.7116	0.4495	0.7879	0.017*	
N23	−0.9455 (4)	0.44088 (14)	0.8943 (3)	0.0129 (6)	
C24	−1.0544 (5)	0.47467 (19)	0.9809 (4)	0.0181 (8)	
H24	−1.1646	0.4523	1.0134	0.022*	
C25	−1.0052 (6)	0.54219 (19)	1.0225 (4)	0.0197 (8)	
H25	−1.0843	0.5653	1.0803	0.024*	
C26	−0.8391 (5)	0.57487 (18)	0.9779 (4)	0.0185 (8)	
H26	−0.8039	0.6200	1.0063	0.022*	
O27	0.6905 (4)	0.80524 (12)	0.0790 (2)	0.0142 (5)	
O28	0.7580 (4)	0.73351 (12)	−0.1150 (3)	0.0204 (6)	
O29	0.4479 (4)	0.80304 (13)	−0.1355 (2)	0.0198 (6)	
O30	0.4820 (4)	0.70339 (12)	0.0130 (3)	0.0231 (6)	
O31	1.0881 (4)	0.72757 (13)	0.1965 (3)	0.0185 (6)	
O32	1.3440 (4)	0.86231 (13)	0.2068 (3)	0.0167 (6)	
O33	1.0745 (4)	0.81868 (13)	−0.0455 (2)	0.0175 (6)	
O34	0.7276 (5)	0.64800 (13)	0.2427 (3)	0.0277 (7)	
O35	0.6380 (5)	0.50133 (16)	0.2090 (3)	0.0393 (8)	
H35	0.6448	0.5437	0.2152	0.059*	
C36	0.4550 (7)	0.4779 (2)	0.2561 (5)	0.0385 (11)	
H36A	0.4866	0.4655	0.3529	0.058*	
H36B	0.4017	0.4380	0.2029	0.058*	
H36C	0.3522	0.5141	0.2446	0.058*	
H31A	1.194 (7)	0.722 (2)	0.251 (4)	0.027*	
H31B	1.000 (7)	0.704 (2)	0.220 (4)	0.027*	
H32A	1.445 (6)	0.846 (2)	0.170 (4)	0.026*	
H32B	1.386 (7)	0.898 (2)	0.215 (4)	0.026*	
H33A	1.198 (7)	0.8143 (19)	−0.063 (4)	0.027*	
H33B	0.995 (6)	0.786 (2)	−0.063 (4)	0.027*	
H34A	0.691 (7)	0.667 (2)	0.320 (5)	0.043*	
H34B	0.615 (7)	0.648 (2)	0.163 (5)	0.043*	

### Atomic displacement parameters (Å^2^)

**Table:**

	*U*^11^	*U*^22^	*U*^33^	*U*^12^	*U*^13^	*U*^23^
Zn1	0.01308 (19)	0.01284 (18)	0.01308 (19)	0.00027 (18)	0.00425 (13)	−0.00021 (18)
S1	0.0139 (4)	0.0144 (4)	0.0138 (4)	−0.0007 (3)	0.0043 (3)	−0.0030 (3)
N1	0.0149 (14)	0.0138 (16)	0.0132 (14)	0.0001 (14)	0.0047 (10)	0.0015 (14)
C2	0.0147 (18)	0.019 (2)	0.0137 (18)	−0.0010 (14)	−0.0003 (14)	0.0010 (13)
C3	0.0111 (17)	0.014 (2)	0.0162 (17)	0.0010 (13)	0.0056 (13)	0.0011 (13)
C4	0.0222 (19)	0.014 (2)	0.0119 (17)	0.0028 (14)	0.0068 (14)	0.0002 (13)
C5	0.0147 (19)	0.0166 (18)	0.0159 (19)	−0.0050 (15)	0.0007 (14)	−0.0001 (15)
C6	0.017 (2)	0.0138 (18)	0.0197 (19)	−0.0057 (15)	0.0074 (15)	−0.0018 (15)
N7	0.0183 (17)	0.0237 (17)	0.0119 (15)	−0.0061 (14)	0.0076 (12)	−0.0029 (13)
C8	0.0115 (18)	0.0167 (19)	0.0166 (19)	0.0038 (14)	0.0046 (14)	0.0024 (15)
O9	0.0190 (14)	0.0218 (13)	0.0133 (13)	−0.0022 (11)	0.0054 (10)	−0.0024 (11)
N10	0.0210 (17)	0.0184 (16)	0.0115 (15)	−0.0037 (13)	0.0073 (12)	−0.0062 (12)
C11	0.0122 (18)	0.0136 (17)	0.0165 (18)	0.0018 (14)	0.0048 (14)	0.0049 (14)
C12	0.0171 (19)	0.020 (2)	0.022 (2)	−0.0037 (16)	0.0133 (15)	−0.0011 (16)
C13	0.031 (2)	0.020 (2)	0.027 (2)	−0.0029 (17)	0.0151 (17)	−0.0104 (16)
C14	0.022 (2)	0.019 (2)	0.017 (2)	−0.0087 (17)	0.0053 (16)	−0.0029 (15)
C15	0.0135 (18)	0.0158 (18)	0.0150 (18)	0.0028 (15)	0.0047 (14)	0.0023 (14)
C16	0.0176 (19)	0.0125 (17)	0.0134 (18)	0.0012 (15)	0.0051 (14)	−0.0008 (14)
N17	0.0162 (16)	0.0114 (15)	0.0239 (17)	−0.0034 (13)	0.0102 (13)	−0.0023 (12)
C18	0.0135 (18)	0.0132 (19)	0.0168 (19)	0.0010 (15)	0.0027 (14)	0.0008 (14)
O19	0.0187 (14)	0.0123 (13)	0.0197 (13)	−0.0020 (10)	0.0051 (10)	−0.0012 (10)
N20	0.0152 (16)	0.0118 (15)	0.0206 (16)	−0.0075 (12)	0.0069 (12)	−0.0046 (12)
C21	0.0097 (17)	0.0181 (19)	0.0159 (19)	−0.0002 (14)	0.0032 (13)	0.0028 (15)
C22	0.0135 (18)	0.0140 (18)	0.0159 (18)	0.0023 (15)	0.0063 (14)	0.0017 (15)
N23	0.0130 (15)	0.0109 (14)	0.0150 (15)	−0.0010 (12)	0.0026 (12)	−0.0008 (12)
C24	0.0160 (19)	0.0206 (19)	0.0182 (19)	−0.0010 (16)	0.0041 (15)	0.0009 (16)
C25	0.020 (2)	0.0181 (19)	0.023 (2)	−0.0019 (16)	0.0102 (16)	−0.0044 (16)
C26	0.019 (2)	0.0146 (18)	0.023 (2)	−0.0004 (16)	0.0061 (15)	−0.0043 (15)
O27	0.0123 (12)	0.0171 (12)	0.0134 (12)	−0.0012 (10)	0.0022 (9)	−0.0033 (10)
O28	0.0161 (13)	0.0223 (13)	0.0242 (14)	−0.0026 (11)	0.0076 (10)	−0.0102 (11)
O29	0.0218 (14)	0.0253 (13)	0.0132 (13)	0.0031 (11)	0.0055 (10)	−0.0013 (10)
O30	0.0283 (15)	0.0196 (14)	0.0234 (15)	−0.0081 (12)	0.0100 (11)	−0.0030 (11)
O31	0.0189 (15)	0.0168 (14)	0.0195 (14)	0.0026 (11)	0.0018 (11)	0.0012 (11)
O32	0.0122 (13)	0.0132 (12)	0.0259 (15)	−0.0036 (10)	0.0070 (10)	−0.0049 (11)
O33	0.0133 (13)	0.0229 (17)	0.0177 (13)	−0.0032 (11)	0.0073 (10)	−0.0038 (10)
O34	0.0349 (17)	0.0226 (15)	0.0268 (16)	0.0016 (13)	0.0088 (13)	−0.0024 (12)
O35	0.0384 (19)	0.0336 (17)	0.050 (2)	0.0036 (15)	0.0193 (15)	0.0041 (15)
C36	0.031 (3)	0.039 (3)	0.049 (3)	0.003 (2)	0.015 (2)	0.010 (2)

### Geometric parameters (Å, °)

**Table:**

Zn1—N1	2.116 (3)	C14—H14	0.9300
Zn1—N23^i^	2.126 (3)	C15—C16	1.383 (5)
Zn1—O33	2.062 (2)	C15—N17	1.416 (4)
Zn1—O31	2.149 (3)	C16—H16	0.9300
Zn1—O32	2.158 (3)	N17—C18	1.373 (4)
Zn1—O27	2.217 (2)	N17—H17	0.8600
S1—O30	1.468 (3)	C18—O19	1.227 (4)
S1—O29	1.471 (2)	C18—N20	1.379 (4)
S1—O28	1.474 (3)	N20—C21	1.406 (5)
S1—O27	1.482 (2)	N20—H20	0.8600
N1—C6	1.331 (4)	C21—C26	1.386 (5)
N1—C2	1.344 (4)	C21—C22	1.392 (5)
C2—C3	1.399 (4)	C22—N23	1.342 (4)
C2—H2	0.9300	C22—H22	0.9300
C3—C4	1.384 (4)	N23—C24	1.346 (5)
C3—N7	1.397 (4)	N23—Zn1^ii^	2.126 (3)
C4—C5	1.364 (5)	C24—C25	1.384 (5)
C4—H4	0.9300	C24—H24	0.9300
C5—C6	1.399 (5)	C25—C26	1.373 (5)
C5—H5	0.9300	C25—H25	0.9300
C6—H6	0.9300	C26—H26	0.9300
N7—C8	1.367 (4)	O31—H31A	0.81 (4)
N7—H7	0.8600	O31—H31B	0.79 (4)
C8—O9	1.230 (4)	O32—H32A	0.85 (4)
C8—O9	1.230 (4)	O32—H32B	0.73 (4)
C8—N10	1.363 (4)	O33—H33A	0.85 (4)
N10—C11	1.409 (4)	O33—H33B	0.82 (4)
N10—H10	0.8600	O34—H34A	0.90 (5)
C11—C12	1.384 (5)	O34—H34B	0.98 (5)
C11—C16	1.394 (5)	O35—C36	1.409 (5)
C12—C13	1.375 (5)	O35—H35	0.8200
C12—H12	0.9300	C36—H36A	0.9600
C13—C14	1.389 (5)	C36—H36B	0.9600
C13—H13	0.9300	C36—H36C	0.9600
C14—C15	1.382 (5)		
			
O33—Zn1—N1	175.25 (12)	C12—C13—H13	118.6
O33—Zn1—N23^i^	89.98 (10)	C14—C13—H13	118.6
N1—Zn1—N23^i^	94.71 (12)	C15—C14—C13	118.1 (3)
O33—Zn1—O31	87.88 (10)	C15—C14—H14	121.0
N1—Zn1—O31	87.42 (12)	C13—C14—H14	121.0
N23^i^—Zn1—O31	177.64 (11)	C16—C15—C14	120.1 (3)
O33—Zn1—O32	86.97 (10)	C16—C15—N17	116.0 (3)
N1—Zn1—O32	93.83 (10)	C14—C15—N17	123.9 (3)
N23^i^—Zn1—O32	89.81 (10)	C15—C16—C11	120.8 (3)
O31—Zn1—O32	91.08 (10)	C15—C16—H16	119.6
O33—Zn1—O27	86.75 (9)	C11—C16—H16	119.6
N1—Zn1—O27	92.41 (10)	C18—N17—C15	128.3 (3)
N23^i^—Zn1—O27	90.39 (10)	C18—N17—H17	115.8
O31—Zn1—O27	88.50 (10)	C15—N17—H17	115.8
O32—Zn1—O27	173.72 (9)	O19—C18—N17	124.4 (3)
O30—S1—O29	108.84 (15)	O19—C18—N20	124.0 (3)
O30—S1—O28	109.45 (14)	N17—C18—N20	111.6 (3)
O29—S1—O28	109.04 (15)	C18—N20—C21	128.1 (3)
O30—S1—O27	109.73 (14)	C18—N20—H20	115.9
O29—S1—O27	109.25 (14)	C21—N20—H20	115.9
O28—S1—O27	110.50 (14)	C26—C21—C22	118.3 (3)
C6—N1—C2	119.2 (3)	C26—C21—N20	117.1 (3)
C6—N1—Zn1	120.1 (2)	C22—C21—N20	124.6 (3)
C2—N1—Zn1	120.7 (2)	N23—C22—C21	122.0 (3)
N1—C2—C3	122.0 (3)	N23—C22—H22	119.0
N1—C2—H2	119.0	C21—C22—H22	119.0
C3—C2—H2	119.0	C22—N23—C24	119.4 (3)
C4—C3—N7	118.1 (3)	C22—N23—Zn1^ii^	121.4 (2)
C4—C3—C2	117.6 (3)	C24—N23—Zn1^ii^	119.0 (2)
N7—C3—C2	124.3 (3)	N23—C24—C25	121.1 (3)
C5—C4—C3	120.8 (3)	N23—C24—H24	119.4
C5—C4—H4	119.6	C25—C24—H24	119.4
C3—C4—H4	119.6	C26—C25—C24	119.7 (3)
C4—C5—C6	118.2 (3)	C26—C25—H25	120.1
C4—C5—H5	120.9	C24—C25—H25	120.1
C6—C5—H5	120.9	C25—C26—C21	119.4 (3)
N1—C6—C5	122.1 (3)	C25—C26—H26	120.3
N1—C6—H6	118.9	C21—C26—H26	120.3
C5—C6—H6	118.9	S1—O27—Zn1	132.11 (14)
C8—N7—C3	127.4 (3)	Zn1—O31—H31A	113 (3)
C8—N7—H7	116.3	Zn1—O31—H31B	117 (3)
C3—N7—H7	116.3	H31A—O31—H31B	108 (4)
O9—C8—N10	123.7 (3)	Zn1—O32—H32A	127 (3)
O9—C8—N10	123.7 (3)	Zn1—O32—H32B	126 (3)
O9—C8—N7	123.8 (3)	H32A—O32—H32B	95 (4)
O9—C8—N7	123.8 (3)	Zn1—O33—H33A	121 (2)
N10—C8—N7	112.4 (3)	Zn1—O33—H33B	97 (3)
C8—N10—C11	127.6 (3)	H33A—O33—H33B	118 (4)
C8—N10—H10	116.2	H34A—O34—H34B	113 (4)
C11—N10—H10	116.2	C36—O35—H35	109.5
C12—C11—C16	119.5 (3)	O35—C36—H36A	109.5
C12—C11—N10	123.4 (3)	O35—C36—H36B	109.5
C16—C11—N10	117.0 (3)	H36A—C36—H36B	109.5
C13—C12—C11	118.6 (3)	O35—C36—H36C	109.5
C13—C12—H12	120.7	H36A—C36—H36C	109.5
C11—C12—H12	120.7	H36B—C36—H36C	109.5
C12—C13—C14	122.8 (4)		

Symmetry codes: (i) −*x*, *y*+1/2, −*z*+1;  (ii) −*x*, *y*−1/2, −*z*+1.

### Hydrogen-bond geometry (Å, °)

**Table:**

*D*—H···*A*	*D*—H	H···*A*	*D*···*A*	*D*—H···*A*
N7—H7···O29^iii^	0.86	2.14	2.954 (4)	157
N10—H10···O29^iii^	0.86	2.25	3.044 (4)	154
N17—H17···O28^iv^	0.86	2.05	2.875 (4)	160
N20—H20···O30^iv^	0.86	2.08	2.922 (4)	164
O35—H35···O34	0.82	2.09	2.893 (4)	168
O32—H32A···O27^v^	0.85 (4)	2.09 (4)	2.942 (3)	179 (4)
O31—H31A···O9^v^	0.81 (4)	2.09 (4)	2.899 (4)	173 (4)
O33—H33A···O29^v^	0.85 (4)	1.87 (4)	2.714 (4)	170 (4)
O34—H34A···O9	0.90 (5)	2.08 (5)	2.861 (4)	146 (4)
O32—H32B···O19^vi^	0.73 (4)	2.08 (4)	2.774 (3)	158 (5)
O31—H31B···O34	0.79 (4)	2.11 (4)	2.887 (4)	168 (4)
O33—H33B···O28	0.82 (4)	1.84 (4)	2.633 (3)	162 (4)
O34—H34B···O30	0.98 (5)	1.90 (5)	2.748 (4)	144 (4)

Symmetry codes: (iii) *x*, *y*, *z*+1; (iv) *x*−1, *y*, *z*+1;  (v) *x*+1, *y*, *z*; (vi) −*x*+1, *y*+1/2, −*z*+1.
